# 

**Published:** 1905-10

**Authors:** 


SUBSCRIPTION $1.00.

PUBLISHED MONTHLY.

### ATLANTA JOURNAL-RECORD OF MEDICINE.

SURGEON GENERAL'S OFFICE
OCT. 11 1905

Successor to Atlanta Medical and Surgical Journal, Established 1855,
and Southern Medical Record, Established 1870.

VOL. VII. ATLANTA, GA., OCTOBER, 1905. No. 7.

